# Effects of Physical Training on the Myocardium of Oxariectomized LDLr Knockout Mice: MMP 2/9, Collagen I/III, Inflammation and Oxidative Stress

**DOI:** 10.5935/abc.20190223

**Published:** 2020-01

**Authors:** Ledimar Brianezi, Elisabete Ornelas, Flávia de Sousa Gehrke, Fernando Luiz Affonso Fonseca, Beatriz da Costa Aguiar Alves, Luiz Vinicius de Alcantara Sousa, Jessica Souza, Laura Beatriz Mesiano Maifrino

**Affiliations:** 1 Laboratório de Estudos Morfoquantitativo e Imunohistoquímico, Universidade São Judas Tadeu, São Paulo, SP - Brazil; 2 Departamento de Farmácia, Universidade Paulista, São Paulo, SP - Brazil; 3 Pós-Graduação em Ciências da Saúde, Hospital do Servidor Público Estadual, São Paulo, SP - Brazil; 4 Laboratório de Análises Clínicas da Faculdade de Medicina do ABC, Santo André, SP - Brazil; 5 Departamento de Ciências Farmacêuticas da Universidade Federal de São Paulo, Diadema, SP - Brazil

**Keywords:** Coronary Artery Disease, Exercise, Menopause, Dyslipidemias, Motor Activity, Collagen, Oxidative Stress, Inflammation, Mice

## Abstract

**Background:**

The emergence of coronary heart disease is increased with menopause, physical inactivity and with dyslipidemia. Physical training is known to promote the improvement of cardiovascular functions.

**Objective:**

To investigate the effects of aerobic physical training on the left ventricle in ovariectomized LDL knockout mice.

**Methods:**

Thirty animals were divided into 6 groups (n = 5): Sedentary non-ovariectomized control; Sedentary ovariectomized control; Trained ovariectomized control; Sedentary non-ovariectomized LDL-knockout, sedentary ovariectomized LDL-knockout and trained ovariectomized LDL-knockout. We analyzed the average parameters of apparent density of collagen fibers types I and III, and metalloproteinase type 2 and type 9, were considered significant p < 0.05.

**Results:**

The results showed that the proposed exercise protocol altered the volume of type I collagen fibers, altered collagen remodeling parameters (MMP-2), and also reduced the 8-hydroxy-2’-deoxyguanosine (8OHdG) oxidative stress parameter.

**Conclusion:**

Moderate intensity aerobic training acts on collagen fiber volume, on collagen remodeling with the reduction of oxidative stress in the left ventricles of ovariectomized LDL-knockout mice.

## Introduction

During the aging process, menopausal women are at increased risk of developing conditions such as dyslipidemia, hypertension, insulin resistance and changes in body composition, where lifestyle and sedentarism are associated with a higher prevalence of the development of cardiovascular disease (CD).^1,2^

The aging process is associated with increased oxidative stress resulting in damage of several cell macromolecules, partly due to decreased antioxidant capacity as well as reduced repair capacity, resulting in increased susceptibility to apoptosis.^3,4^ Particularly in menopausal women, neuroendocrine alterations affect the functionality, metabolic capacity and antioxidant activity of numerous organs, especially due to the lack of estrogen, considered a female antioxidant, resulting in an additional increase of oxidative stress.^5^

Lipid metabolism is also influenced by physiological changes during menopause resulting in an increase in LDL and a decrease in HDL and contributes to the emergence of cardiovascular diseases.^6,7^ When compared to men of the same age, postmenopausal women are at an increased risk of developing heart disease.^8,9^ It is one of the main causes of morbidity and mortality in this physiological stage.^10^

Regular physical activity relieves the effects of aging and menopause and improves aerobic fitness, maintaining body weight index of visceral fat, plasma lipid levels, increased insulin sensitivity, increased baroreflex sensitivity and improved endothelial function, capillary wall shear stress which results in increased blood flow, stimulating nitric oxide release.^11-13^ These factors promote a better health-related quality of life and prolong survival and can be considered essential non-pharmacological standards in the treatment of postmenopausal effects and other physiological and pathological conditions.^14,15^

The objective of this study is to analyze the effects of aerobic physical training on the left ventricle in ovariectomized wildtype and LDLr knockout female mice on the following parameters: volume density of types I and III collagen fibers, metalloproteinases type 2 and type 9 expression, in addition to COX2 and 8-OhdG expression.

## Methods

### Animals

Thirty female mice aged 10 months were used: 15 genetically modified female mice, with knockout of the low-density lipoprotein receptor (LDLr Knockout), and 15 female wildtype mice (C57BL/6J) obtained from the University of São Paulo vivarium. The animals were kept in a USJT vivarium at a controlled temperature (22-24°C) and lighting (12 hours of light cycle and 12 hours of dark) receiving commercial feed (NUVILAB CR1, Nuvital Nutrients LTDA, Curitiba, PR) and water "ad libitum". The animals were divided into 6 groups (n = 5): sedentary non-ovariectomized control (CS), sedentary ovariectomized control (COS); trained ovariectomized control (COT); non-sedentary ovariectomized LDL knockout (LDL-S), sedentary ovariectomized LDL knockout (LDL-OS) and trained ovariectomized LDL knockout (LDL-OT). The division of the animals in the groups was performed by convenicence.

The experimental protocol was approved by the Research Ethics Committee of Universidade São Judas Tadeu (CEP-Protocol: 058/2007) and the research was conducted according to the Principles of Laboratory Animal Care formulated by the National Institutes of Health.

### Ovariectomy

At nine months of age animals underwent the ovariectomy procedure. The animals were anesthetized with a ketamine and xylazine solution (120:20 mg/Kg, im) and placed in supine position and a small incision in the lower third of the abdominal region, parallel to the line of the body, was made. The ovaries, the uterine horns, and the blood vessels were located, sectioned, and removed. After that, the musculature and the skin were sutured. Confirmation of the efficacy of the ovariectomy was determined through colpocytology of the vaginal secretion performed over four consecutive days. On the last day of analysis, euthanasia was performed on these animals.^16^

### Training Protocol

#### Maximal training Test

A maximal training test was performed on all the groups at the beginning and at the end of the exercise training program. The test consists of placing the animal to run on an ergometric treadmill at 0.3 km/h for 3 minutes, and this workload was increased by 0.3 km/h every 3 minutes until the animal reached exhaustion. The time of the test and the speed of the last workload were noted and served to determine the mean value of aerobic capacity of each group.

#### Exercise training

Exercise training began 7 days after the ovariectomy surgery; the trained groups were subjected to a physical training protocol on an ergometric treadmill at low-moderate intensity (≈50% to 70% maximal running speed) for 1 hour a day, 5 days a week, for 4 weeks, with a gradual increase in speed from 0.3 to 1.2 km/h. The animals were adapted to the treadmill for 10 minutes on three days prior to beginning the training.

#### Euthanasia and tissue preparation

At the end of the training the animals were sacrificed by decapitation. A thoracotomy was performed in which the heart and atria were removed and the right and left ventricles were sectioned. Left ventricle samples were fixed in 10% buffered formalin for 24 hours. Afterwards, the tissue was transferred to a 70% ethyl alcohol solution, dehydrated in increasing ethanol series, diaphanized in xylol and embedded in paraffin. Non-serial, 5 µm thick cuts were performed in which each section received a total of 6 cuts. The sections were stained with the Picrosirius technique, for the analysis of collagen fibers I and III in the left ventricle and visualized by polarized light microscopy.

The volume density of collagen fibers I and III (Vv[fc]) expresses the fraction of the volume occupied by the collagen fibers in relation to the total volume of the analyzed image. For this analysis, the test system was used with a total of 475 points, which corresponds to 100% of the image, using the program Image J. (version 1.47 - National Intitutes of Health-(Collins).

#### Immunohistochemistry

For the immunohistochemical techniques, 3 to 4 micrometer thick cuts were made and mounted on slides previously silanized using 4% silane. After the slides were dewaxed, using a heating oven overnight set at 57ºC, after which they were immersed in xylol baths for 30 minutes. Afterwards, they were hydrated in ethyl alcohol with decreasing concentrations of 100%, 80% and 70%, each with a duration of 5 minutes, then washed in running water. In the next step, the antigenic recovery was performed in a water bath at 90°C, the slides were placed in citrate buffer for 30 minutes, then washed in running water. Endogenous peroxidase blockade was performed in H_2_O_2_ for 15 minutes, followed by washing in distilled running water and in PBS pH = 7.4. For each slide, a specific antibody was used, table 1, and the antibody was placed overnight in a humid chamber at 4°C. The material was washed with PBS buffer and incubated with a biotinylated secondary antibody for 30 minutes, washed again with PBS and incubated with a streptoavidin-peroxidase antibody for another 30 minutes. A chromogenic substrate, DAB (33-Diaminobenzidine) solution in the ratio of 1:1 were used for five minutes at room temperature to wash with PBS solution and to reveal the reaction. When the presence of a dark brown precipitate was observed the slides were placed in running water, after which they were counterstained with "Hematoxylin Harrys" for 2 minutes. Afterwards, they were submitted to 3 xylol baths to diafieze and to 2 alcohol baths. The slides were mounted with coverslips and Entellan®.

**Table 1:** Antibodies used for protein detection

Antibody	Dilution	Labeling	Function	Manufacturer
MMP2	1:250	Cytoplasm	Collagen remodelling	sc-10736, Santa Cruz®
MMP9	1:300	Cytoplasm	Collagen remodelling	sc-6840, Santa Cruz®
COX 2	1:100	Cytoplasm	Inflammation	sc-1745 P, Santa Cruz®
8-OHdG	1:100	Cytoplasm	Oxidative stress	sc-66036, Santa Cruz®

**Figure 1:**
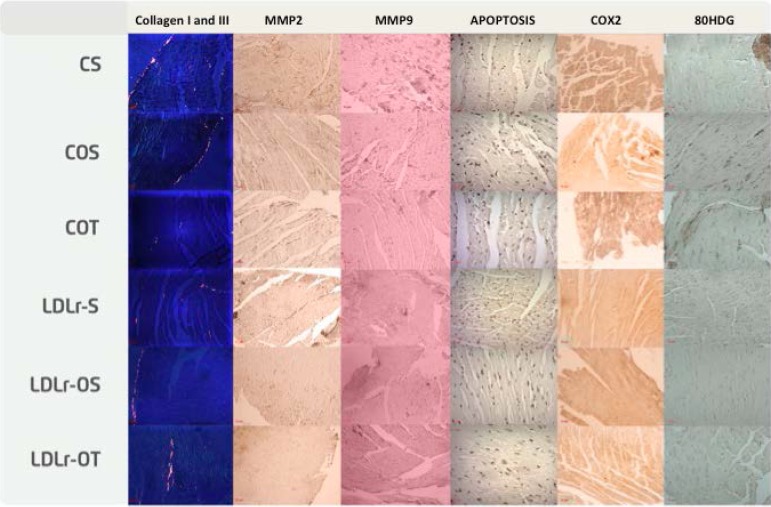
Immunoexpression of proteins. CS: sedentary non-ovariectomized control; COS: sedentary ovariectomized control; COT: trained ovariectomized control; LDLr-S non-sedentary ovariectomized knockout; LLr-OS: sedentary ovariectomized LDL knockout; LDLr-OT: trained ovariectomized LDL knockout.

In all the techniques, observing the presence of a dark brown precipitate, the sample was visualized under an optical microscope. A quantitative analysis of the images was performed using the program ImageLab 2000, where the brown markers were selected and the program quantified immunoexpression intensity, figure 1.

### Statistical analysis

Absolute and relative values were used to describe the qualitative variables. For the quantitative variables with normal distribution (Shapiro-Wilk > 0.05), mean, standard deviation, minimum and maximum were used, and for the variables with non-normal distribution (Shapiro-Wilk < 0.05), median and percentiles were used. To study the diferences between the variables with the groups, the Kruskal-Wallis and ANOVA tests were used, complemented by the Dunn test or Bonferroni. For all analyzes, a confidence level of 95% was used. The program utilized for these analyzes was Stata version 11.0. (StataCorp. 2009. *Stata Statistical Software: Release 11*. College Station, TX: StataCorp LP.)

## Results

Our data show a significant reduction in the volume density of type I collagen fibers in the ovariectomized dyslipidemic group (LDL-OS) (-51%). The LDL-OT group showed a decrease of 100% when compared to the COS group and a significant difference in relation to the COT and LDL-S groups. Table 2 shows the association of COX-2 and MMP9 expression variables and the density of type I collagen fibers in relation to the evaluated animal group.

**Table 2:** Comparison of Cyclooxygenase-2, Volume Density of collagen fibers I and Metalloproteinase type 9, with the groups studied. clinical variables with groups

Variables	CS	COS	COT	LDL-S	LDL-OS	LDL-OT	p*
COX-2	35.0 (23.9; 46.3)	46.5 (44.2; 79.7)	46.1 (31.3; 47.7)	47.7 (45.8; 63.1)	87.8 (42.5; 92.4)	49.4 (49.1; 50.9)	0.232
Vv [fc I]	0.21 (0.21; 0.43)	0.43 (0.21; 0.43)	-	-	0.21 (0.0; 0.41)^A^^,^^B^^,^^C^	0.0 (0.0; 0.21)^A^^,^^B^^,^^C^	< 0.001
MMP9	44.7 (40.1; 44.8)	40.9 (39.8; 45.6)	53.9 (48.6; 66.2)	53.0 (38.3; 58.1)	42.2 (27.3; 53.7)	64.1 (45.5; 93.9)	0.169

* Kruskal-Wallis. Data expressed as median and 25% and 75% percentiles. COX-2: Cyclooxygenase-2; Vv [fc I]: Volume Density of collagen fibers I; MMP 9: Metalloproteinase type 9; CS: sedentary non-ovariectomized control; COS: sedentary ovariectomized control; COT: trained ovariectomized control; LDL-S: non-sedentary ovariectomized LDL knockout; LDL-OS: sedentary ovariectomized LDL knockout; LDL-OT: trained ovariectomized LDL knockout.

A statistically significant difference between the studied group and the COS group;

B statistically significant difference between the studied group and the TOC group;

C statistically significant difference between the studied group and the LDL-S group.

Regarding metalloproteinase type 2 expression, in the control group (CS) there was a significant increase of 24% in the ovariectomized group (COS), when physical training (COT) was performed there was an increase of 72%, and with dyslipidemia (LDL-S), the increase was 92%. In the dyslipidemic group with ovariectomy (LDL-OS), the values decrease 27% in relation to the LDL-S group and 18% in relation to the COT group, but still above the values of the control groups by 42% in relation to the CS group and 14% in relation to the COS group. When the training was performed in the ovariectomized dyslipidemic group LDL-OT, the results obtained present a 15% increase in relation to the sedentary ovariectomized LDL-OS group and a 15% decrease in relation to the LDL-S group. When compared to the control group, there was an increase of 64% in relation to the CS group, 32% in relation to the COS group and a decrease of 5% in relation to the COT group.

When analyzing the oxidative stress data, evaluating 8OHdG expression, there was a decrease in the indexes for the control group (CS), in which there was a decrease of 33% in the group with ovariectomy (COT) and a 39% decrease in the trained group. When dyslipidemia was present (LDL-S), there was a 51% decrease in relation to the CS group, and in the dyslipidemic group with ovariectomy (LDL-OS) there was a decrease of 85% in relation to the CS group, 78% in relation to the COS group, 76% in relation to the COT group and 70% in relation to the LDL-S group. When training was performed in the dyslipidemic ovariectomized group (LDL-OT), the data presented a 152% increase in relation to the LDL-OS group and a decrease in relation to the control groups, with 63% for the CS group, 45% for the COS group and 40% for the COT group. For the parameters related to volume density of type III collagen fibers, metalloproteinase type 9 and inflammation (COX2), the values obtained for the control and dyslipidemic groups do not present significant differences in any parameter, table 3.

**Table 3:** Comparison of the variables Metalloproteinase type 2, Anti-8-Hydroxydeoxyguanosine and Volume Density of collagen fibers III (Vv [fc III]), with the groups studied.

Variables	CS	COS	COT	LDL-S	LDL-OS	LDL-OT	p*
MMP 2	28.5 (4.0)	35.3 (9.1)^A^	49.0 (3.8)^A^	54.8 (52.0)^A^	40.5 (9.6)^A^^,^^B^^,^^C^^,^^D^	46.8 (31.0)^A^^,^^B^^,^^C^^,^^E^	< 0.001
8-OHDg	105.2 (7.6)	70.0 (10.8)^A^	64.9 (4.7)^A^	51.7 (0.5)^A^	15.3 (7.5)^A^^,^^B^^,^^C^^,^^D^	38.7 (8.4)^A^^,^^B^^,^^C^^,^^E^	< 0.001
Vv[fc III]	0.007 (0.037)	0.003 (0.024)	0.016 (0.067)	0.015 (0.057)	0.013 (0.062)	0.009 (0.043)	0.649

* ANOVA. Data expressed as mean and standard deviation.

A statistically significant difference between the studied group and the CS group;

B statistically significant difference between the studied group and the COS group;

C statistically significant difference between the studied group and the TOC group;

D statistically significant difference between the studied group and the LDL-S group;

E statistically significant difference between the studied group and the LDL-OS group.

MMP2: Metalloproteinase type 2; 8OHdG: Anti-8-Hydroxydeoxyguanosine; Vv [fc III]: Volume Density of collagen fibers III; CS: sedentary non-ovariectomized control; COS: sedentary ovariectomized control; COT: trained ovariectomized control; LDL-S: non-sedentary ovariectomized LDL knockout; LDL-OS: sedentary ovariectomized LDL knockout; LDL-OT: trained ovariectomized LDL knockout.

## Discussion

Physical activity is recognized as an important non-pharmacological treatment for numerous diseases and conditions, including dyslipidemia and menopause. Studies indicate that collagen fibers are present in the process of myocardial remodeling, which occur due to aging and other factors.^17,18^

Our data show that in the myocardium, the expression of type III collagen fibers did not differ between groups and parameters. In relation to type I collagen, ovariectomy and training brought a decrease in the levels presented in relation to the sedentary ovariectomized control group. Results similar to those found in our study verified the same variable in animals after a period of obesity induced by a diet rich in unsaturated fat.^19^ Interestingly, the findings reported in this article have similar results only with the proposed physical activity. Another study where acute myocardial infarction was induced in rats, the gradual increase of types I and III collagen was observed at 4 weeks. The kinetics of collagen I/III increase, in combination with the decrease of the elastic fibers in the infarcted area after an MI, provided evidence that impaired cardiac function after an MI was due to healing or infarct scar formation, with increased rigidity and less flexibility of the heart.^20^

Our results suggest that the aging period in this study does not appear to interfere with the increase in the expression of collagen fibers presented in other studies.^21,22^ However, when evaluating the LDL-OT groups, there is a clear reduction in the volume of type I collagen fibers. This verification was obtained using the proposed training protocol (intensity or duration).^23,24^

Metalloproteinase type 2 and type 9 also participate in the remodeling process of cardiac tissue, and are present in several pathologies such as inflammatory and cardiovascular diseases, among other injuries.^24-29^ The expression of metalloproteinase type 9 did not differ between groups and parameters. Regarding metalloproteinase type 2, an increase was shown in the control group with ovariectomy and physical training even in the presence of dyslipidemia. This process explains the factor with the collagen indexes when these are not elevated, because the degradation of collagen and the extracellular matrix is peformed by the action of the metalloproteinases.^25,30-32^ The elevated presence of MMPs in patients with dyslipidemias found in some studies suggest their participation in the matrix degradation process in atherosclerotic plaques and in the ruptures of these and with exposure of the nucleus^17,33^ and are independent predictors for the progression of kidney disease and are independently associated with an increased risk of mortality.^34^

The expression of COX2 in our study did not present statistical significance, indicating that in all groups and parameters, there were no changes for these data. Thus, in this parameter chosen to verify inflammation, no changes are observed showing that the factors associated with the groups do not interfere in such an evaluation.

Aging, menopause and dyslipidemia are factors that also result in oxidative stress.^3-5^ In our research, oxidative stress was verified through the expression of 8OHdG, and ovariectomy decreased the values of this parameter in the control and dyslipidemic groups, differing from the results obtained in other studies.^5,35^ When physical training was performed, the values were decreased in the control group, but in the dyslipidemic group, they presented an increase. Interestingly, this marker seems to have no association with the groups proposed in this experimental model.

## Conclusion

The data of this research indicate that physical exercise beneficially influenced the control and dyslipidemic groups in the volume density parameter of type I collagen fibers and the control group in relation to oxidative stress.
